# Transactions of the Twenty-Fourth Anniversary Meeting of the Illinois State Medical Society, Held in the City of Chicago, May 19, 20 and 21, 1874

**Published:** 1875-01-15

**Authors:** 


TRANSACTIONS OF THE TWENTY-FOURTH ANNIVERSARY MEETING OF THE ILLINOIS STATE MEDICAL SOCIETY, held in the City of Chicago, May 19, 20 and 21, 1874. Chicago: Fergus Printing Company, 244–248 Illinois Street. 1874.

This is a volume of 248 pages, printed in good style. It contains a full record of the proceedings of the last annual meeting of the Illinois State Medical Society, including the papers read and the discussions relating to the same, together with the list of officers and members. As a whole, the volume is highly creditable to the Society, and to the officers under whose direction it was issued; and yet it is marred by certain important defects, that certainly should be guarded against in the future.

It is the second year of the experiment of employing a professional reporter, to furnish full reports of all verbal or extempore remarks by officers and members during the annual meetings. While the result has shown that such reports, if properly revised while passing through the press, add very much to the interest and value of the transactions, it has equally shown, that without such revision, every member who speaks is liable to have, either his thoughts so jumbled by the reporter that he can hardly recognize them as his own, or his words spelled wrong, or both. When matter is hurried through the press for a daily newspaper, there may be excuse for neglecting revision, and careful proof reading; but when four or five months are occupied in putting a work of 200 or 300 pages through the press, there can be no reasonable excuse for making *mite* read "might;" expectant, "expectorant;" simple continued fever, "self-continued;" alimentary canal, "abdominal canal," etc.; or to send out to the world such unmitigated nonsense as the following:

"We should aim to adapt the *nutritive* capacity of the patient to it, (the food) in such shape as to require the least change, particularly if the tongue and alimentary canal are coated. Why? Because something foreign has got there that must be got out. It is because there is a derangement of the *efficiency* of the secreting cells of the mucous membrane, so that they fail to secrete the normal amount of *lubricating material*."

The time was amply sufficient to have given every member whose remarks were reported, an opportunity to have revised, or at least read the proof sheets, of the matter attributed to him; and in future, the Society must require this to be done, or else follow the example of the American Medical Association, and employ no one as reporter, who is not also an educated physician: for abundant experience has shown, that no stenographer, however skillful, can report medical lectures or discussions correctly, unless he has also a good knowledge of medical science. We make these remarks, not from any disposition to find fault with the committee of publication, but from a sense of duty, and with the hope of securing better results in the future. N. S. D.

